# Functionalization of zeolite-encapsulated Cu_5_ clusters as visible-light photoactive sub-nanomaterials†

**DOI:** 10.1039/d4ra08633c

**Published:** 2025-01-22

**Authors:** Katarzyna M. Krupka, Lenard L. Carroll, María Pilar de Lara-Castells

**Affiliations:** a Institute of Fundamental Physics (AbinitSim Unit, ABINITFOT Group), Consejo Superior de Investigaciones Científicas (CSIC) E-28006 Madrid Spain Pilar.deLara.Castells@csic.es

## Abstract

The unique structural properties of zeolites make them ideal environments for encapsulating subnanometric metal clusters on their microporous channels and cavities, showing an enhanced catalytic performance. As a first step towards the functionalization of these clusters as photocatalysts as well, this work addresses the optical properties of zeolite-encapsulated Cu_5_–TiO_2_ nanoparticles as well as their application in the photo-induced activation of CO_2_ by sunlight. Model density functional theory (DFT) calculations indicate the stability of the Cu_5_ cluster adsorbed on the TiO_2_ nanoparticles filling the pores of a model zeolite structure. Second, it is shown that while TiO_2_ nanoparticles absorb in the UV, the photo-absorption spectrum of the Cu_5_–TiO_2_ nanoparticle composite is peaked at the visible region, where the sun has its maximum energy input, also allowing for the photo-induced activation of CO_2_ adsorbed onto the Cu_5_ cluster.

## Abstract 2

While TiO_2_ nanoparticles absorb in the UV of the solar spectrum, it is shown that zeolite-encapsulated Cu_5_–TiO_2_ nanoparticles absorb in the visible region *via* direct photo-induced electron transfers and formation of charge-separated states.

## Graphical abstract

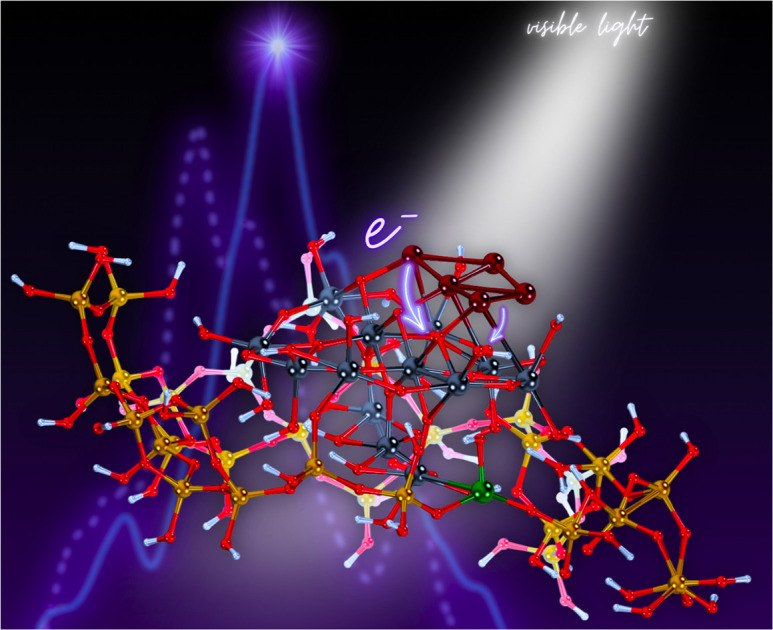

## 1 Introduction

With the advent of the subnanometer scale as a critical feature size, our chemical and physical insight into the structure of matter is deepening almost exponentially.^1^ One of the key aspects driving this transformation in materials science is the emergence of highly selective experimental techniques allowing for the synthesis of monodisperse atomically precise metal clusters (referred to as AMCs), composed of just one to tens of atoms.^2^ Exhibiting strong quantum confinement effects, AMCs are attracting significant interest in various fields due to their unexpected stability and unique properties with potential applications in catalysis,^3–10^ photocatalysis,^11–14^ luminescence,^15^ therapeutics,^16^ bioimaging,^17,18^ and sensing,^19^ bridging fundamental science and the world of nanotechnology (see, *e.g.*, ref. 20 for a recent perspective).

Featuring molecular-like structures and discrete energy levels,^21–25^ AMCs, typically smaller than 1 nm in size, differ markedly from larger nanoparticles and isolated metal cations in their catalytic behaviors. These differences are strongly influenced by low coordination numbers, high surface-to-volume ratios, and structural fluxionality, which arises from dynamical fluctuations in their atomic arrangements.^12,13,20,26–33^ As a result, subnanometric metal clusters provide a plethora of dynamically inter-connected active sites, and offer significantly higher reactivity compared to larger nanoparticles.^34–36^

For instance, copper pentamer clusters exhibit novel and remarkable properties, including exceptional thermodynamical and chemical stability in solution across the entire pH range (1–14),^37^ an unexpected resistivity to deep irreversible oxidation despite their tiny size,^32,38–40^ an applicability to different catalytic reactions,^12,41,42^ and their capability to improve the optoelectronic and (photo)catalytic properties of photo-absorbers such as titanium dioxide.^11,43^

The encapsulation of subnanometric metal clusters in porous materials such as zeolites,^25^ carbon nanotubes,^44–46^ and metal–organic frameworks^47,48^ (MOFs) is an effective strategy for stabilizing and controlling their reactivity, offering enhanced catalytic performance and selectivity. By tuning the pore size, shape, and surface chemistry of these materials, AMCs can be effectively immobilized, preventing their aggregation. Furthermore, the high surface area and porosity of zeolites and MOFs facilitate efficient mass transfer and substrate accessibility.^49–51^ For instance, a recent study has demonstrated that heteroatom-doped Ag_25_ clusters encapsulated in MOFs drive photocatalytic hydrogen production.^47^ Moreover, the versatile applications of porphyrin-based metal–organic frameworks (PMOFs) as photocatalysts in aqueous environments have been recently reviewed,^52^ with a specific focus on solar fuel production and pollutants degradation. The use of metal–organic framework heterojunctions for photocatalysis has been also summarized^53^ alongside the application of MOFs as solid catalysts for liquid-phase reactions, including synthesis by design, high porosity in the micro-mesoporous range, and a high density of unsaturated transition-metal ions.^54^ Likewise, it has been shown that the encapsulation of AMCs within carbon nanotubes enhance the catalytic activities of AMCs in various reactions, including Fischer–Tropsch synthesis,^44^ syngas-to-ethanol transformations,^45^ and ester hydrogenation reactions.^55^ Additionally, AMCs are less prone to oxidation.^46^

Zeolites, crystalline aluminosilicates known for their well-defined pore structures, high thermal stability, and tunable acidity and basicity,^56^ are excellent candidates for hosting subnanometric metal clusters.^57,58^ The spatial confinement within the microporous channels and cavities of zeolites stabilizes them, hindering their escape.^59,60^ This stabilization helps in suppressing the migration and aggregation of the metal clusters, as demonstrated in high-temperature oxidation and reduction treatments.^36^ Moreover, as recently reviewed in ref. 25, zeolite-encapsulated AMCs exhibit higher catalytic efficiencies than their non-encapsulated counterparts in various reactions, such as propane dehydrogenation, formic acid decomposition, ammonia borane hydrolysis, cyclohexane oxidation, the water–gas shift reaction, and hydrogenation. These encapsulated clusters also demonstrated higher hydrogen evolution rates.^61^ Remarkably, the encapsulation of AMCs in zeolites has also made it possible to achieve one of the highest turnover frequency values for heterogeneously catalyzed formic acid decomposition.^62^

Naturally, experimental capabilities for synthesizing and characterizing zeolite-encapsulated AMCs have advanced hand-in-hand with those allowing their theoretical underpinnings such as state-of-the-art (single-reference) dispersion-corrected density functional theory (DFT)-based modelling. These developments have enabled the investigation of the catalytic behaviour of subnanometric copper clusters in the methane to methanol (MTM) reaction supported in zeolites and zeotypes using O_2_ as oxidant without water molecules assisting the process.^63–66^ Notably, studies have demonstrated that the selective oxidation of methane to methanol can be achieved with Cu_5_ clusters encapsulated in zeolite structures.^64^ However, while these studies have primarily focused on electronic ground-state properties, there remains a lack of theoretical investigations exploring the excited states and, in particular, the optical properties of these encapsulated systems.

Our work addresses this significant research gap by using advanced methods to investigate the encapsulation of Cu_5_ clusters supported on a TiO_2_ nanoparticle within the micropores of a zeolite structure. This approach allows us to explore not only the fundamental electronic structure but also the photo-absorption spectra in great detail. Our study is motivated by the joint experimental–theoretical demonstration that the deposition of a single monolayer of Cu_5_ on a TiO_2_ surface markedly enhances its optical properties, making it a visible-light photoactive material (see, *e.g.*, ref. 11). As a result, much more energy can be harvested from sunlight, with the coated titanium dioxide temporally storing this energy as charge pairs (*i.e.*, electrons and holes), an ideal prerequisite for follow-up chemistry. Notably, a later study^12^ demonstrated the photoinduced activation of CO_2_ physisorbed on TiO_2_-modified Cu_5_ clusters under visible light. Our work is further inspired by a recent study^67^ that highlighted the successful use of TiO_2_ nanoparticles within MOF mesopores for CO_2_ photoreduction under UV light.^68^ By modeling the same process using Cu_5_-modified TiO_2_ nanoparticles instead, the photoreduction could potentially extend into the visible light spectrum, enhancing its applicability and efficiency.^68^

Applying density functional theory (DFT) and a combined approach that integrates DFT with reduced density matrix theory, we investigate the optical response of the system under solar irradiation. Thus, in Section 2, the computational approach and the details of our calculations are presented. Section 3 focuses on the analysis of the photo-absorption spectra of non-encapsulated and zeolite-encapsulated Cu_5_–TiO_2_ composite nanoparticles, including their potential for physisorbed CO_2_ photo-reduction. Finally, Section 4 closes with the concluding remarks.

## 2 Computational–theoretical methods

### 2.1 Structural models

Following ref. 69, a hydrogen-saturated cluster of stoichiometry Ti_9_O_25_H_14_ has been used as a model for a TiO_2_ nanoparticle based on the experimental geometry of the TiO_2_(110)-(1 × 1) surface.^70^ The structure of the TiO_2_ units within the nanoparticle remains unchanged from the original surface, ensuring it accurately represents the properties of bulk TiO_2_ in nanoparticulate form. The planar trapezoidal two-dimensional (2D) Cu_5_ structure has been selected as the initial configuration (referred to as Cu_5_-2D).

**Fig. 1:**
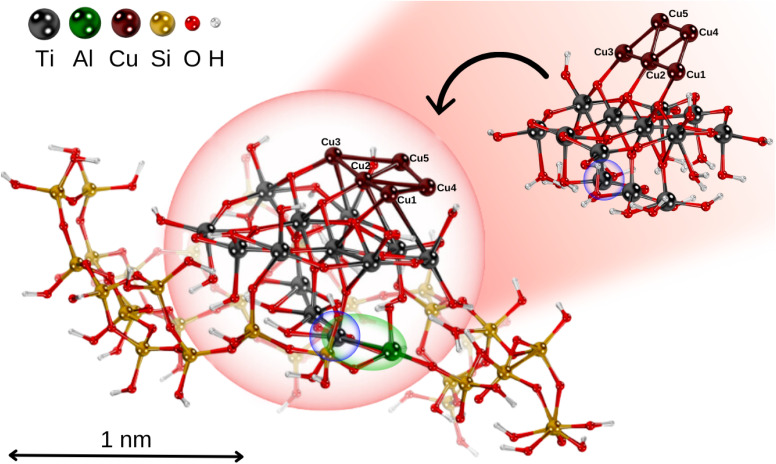
Picture illustrating the structural models of the non-encapsulated Cu_5_–TiO_2_ nanoparticle and the zeolite-encapsulated one. Position of a polaron-like, localized electron on the titanium atom is shown in blue circle; Ti–Al bond highlighted in green.

The TiO_2_ nanoparticle was inserted into a zeolite pore model based on the extra-large-pore zeolite structure UTD-1 from ref. 71, which is constructed from typical tetrahedral SiO_4_ units forming oxygen 4-, 5- and 6-membered rings.^72^ The resulting microporous structure has a diameter close to the size of the TiO_2_ nanoparticle (see Fig. 1). To achieve charge balance and enhance the interaction capabilities, one silicon atom in the zeolite framework was substituted with an aluminum atom, resulting in Si–O–Al linkages that generated a net negative charge in the system.^64,72^ As shown in Fig. 1, the TiO_2_ nanoparticle adheres to the microporous structure of the zeolite framework *via* a Ti–Al bond.

### 2.2 Computational details

Periodic electronic structure calculations were performed using spin-polarized DFT + *U* as implemented in the Vienna *Ab initio* Simulation Package (VASP 5.4.4).^73,74^ The Perdew–Burke–Ernzerhof (PBE) functional was used to approximate the exchange correlation potential.^75^ Geometries were relaxed using the conjugate gradient method, with dispersion corrections applied using the DFT-D3 approach^76,77^ with Becke–Johnson damping. Dipole corrections were incorporated along the *z*-direction in the simulation cell, and particle occupancies were handled using a Gaussian smearing scheme with a smearing factor 0.05 eV. Hubbard U parameters of 4.2 eV for Ti and 5.2 eV for Cu were adopted, consistent with a prior study of the Cu_5_–TiO_2_(110) system.^11^ Electron–ion interactions were described by the projector augmented-wave (PAW) method,^78,79^ using PAW-PBE pseudopotentials (PP) as implemented in VASP. Electrons of O 2s2p, Al 2s2p, Si 2s2p and Cu 3d4s orbitals were explicitly treated as valence electrons. For Ti atoms, two calculations sets were considered: one treating 3d4s and another treating 3s3p3d4s as valence electrons, referred to as 3d4s + PP and 3s3p3d4s + PP. A plane-wave basis set with a kinetic energy cutoff of 400 eV was used, while a cut-off energy of 605.392 eV for the plane wave representation of the augmentation charges was chosen. The Brillouin zone was sampled at the *Γ* point, to minimize computational cost. The convergence threshold criterion was set to 10^−4^ eV for the self-consistent electronic minimization. Geometries were relaxed with a force threshold of 0.02 eV Å^−1^. We selected a cell volume for Cu_5_–TiO_2_@zeolite of 5 × 5 × 5 nm^3^, avoiding the interaction between periodic replicas.

As a complement to the periodic DFT-based study, all-electron cluster model calculations were carried out using the same DFT-D3 (BJ) scheme with the ORCA^80^ suite of programs (versions 5.0.4 and 6.0.0). For this purpose, the atom-centered (augmented) polarized correlation-consistent triple-*ζ* (aug-cc-pVTZ) basis of Woon and Dunning Jr,^81^ as reported in ref. 69 was used for oxygen and titanium atoms.

The Cu_5_–TiO_2_ and Cu_5_–TiO_2_@zeolite interaction energies (*E*_int_) were calculated as,*E*_int_(Cu_5_–TiO_2_) = *E*_Cu_5_–TiO_2__ − *E*_Cu_5_ −_*E*_TiO_2__*E*_int_(Cu_5_–TiO_2_@zeolite) = *E*_Cu_5_–TiO_2_@zeolite_ − *E*_Cu_5__ − *E*_TiO_2_@zeolite_where *E*_Cu_5_−TiO_2__ and *E*_Cu_5_–TiO_2_@zeolite_ are the energies of the Cu_5_–TiO_2_ and Cu_5_–TiO_2_@zeolite molecular complexes at the potential energy minima while *E*_Cu_5__ denotes the energy of the isolated Cu_5_ clusters, adopting the structures present in the Cu_5_–TiO_2_ and Cu_5_–TiO_2_@zeolite complexes. Similarly, *E*_TiO_2__ and *E*_TiO_2_@zeolite_ are the energies of the isolated TiO_2_ and TiO_2_@zeolite systems with the geometries acquired in the same complexes.

Calculations of the interaction potentials between a single Cu atom and a TiO_5_H_6_ nanoparticle were carried out at the DFT-D3 and Møller–Plesset second-order perturbation theory (UMP2) levels using the aug-cc-pVTZ basis set with the ORCA code.^80^ Uncoupled, open-shell Hartree–Fock and coupled dispersion energy contributions were independently obtained in Symmetry-Adapted Perturbation Theory (SAPT) calculations using the Psi4 and Psi4NumPy software packages.^82–85^ For details on the methodology, see ref. 86.

### 2.3 Reduced density matrix treatment

In order to obtain the photo-absorption spectra, we have applied the Reduced Density Matrix (RDM) approach in the Redfield approximation,^87^ using the orbitals obtained in periodic calculations with the HSE06 approach. This RDM-DFT approach was proposed by Micha and collaborators.^88–90^ It has been successfully applied to AMCs adsorbed on semiconductor rutile TiO_2_(110) (see ref. 13 and references cited therein) and silicon surfaces.^91–94^ The performance of the RDM-DFT scheme has been directly evaluated by comparing the absorbance of TiO_2_ and Cu_5_–TiO_2_ interfaces with the experimentally determined one (*via* diffuse reflectance spectroscopy) in ref. 11.

For the sake of completeness, we briefly overview the main equations applied within the RDM-DFT framework (see, *e.g.*, ref. 88 for a detailed description). When the surface of the material is exposed to a monochromatic electromagnetic field 
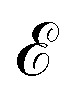
 of frequency *Ω*, the evolution equation for the RDM, referred to as *ρ*, can be expressed as,1
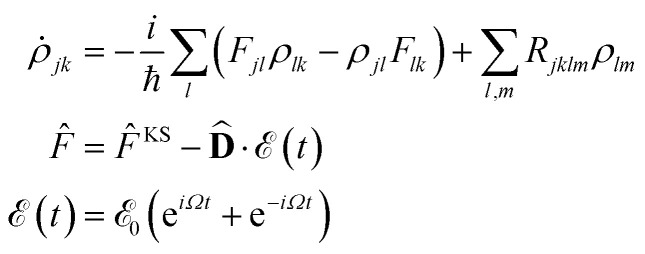
where *F̂*^KS^ is the Kohn–Sham (KS) Hamiltonian, which is represented in the basis set of KS orbitals. In eqn (1), **D̂** is the electric dipole moment operator. Finally. *R*_*jklm*_ are the Redfield coefficients (*i.e.*, the KS component of the relaxation tensor, see ref. 87 and 88 for details). Next, in order to account for the electromagnetic field oscillations, a coordinate transformation is carried out into a rotating frame as,
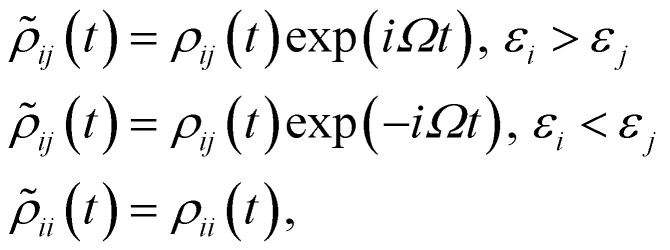
where *ε*_*i*_ is the energy of the *i*th Kohn–Sham orbital. An average over time of the fast terms in the equations of motion of the RDM yields^88,93^
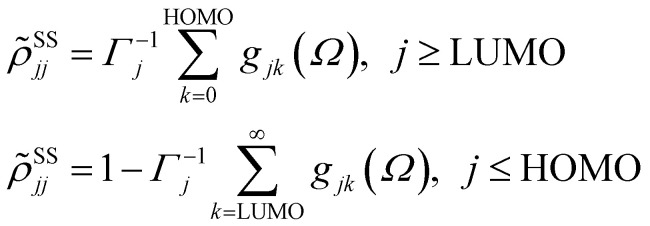
with HOMO (LUMO) denoting the highest-energy occupied (lowest-energy unoccupied) orbitals, *Γ*_*j*_ standing for the depopulation rate. The terms *g*_*jk*_ are expressed through the equation2
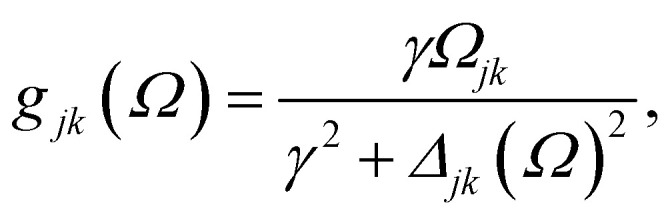
where *γ* is the decoherence rate, and *Ω*_*jk*_ are the Rabi frequencies expressed as 
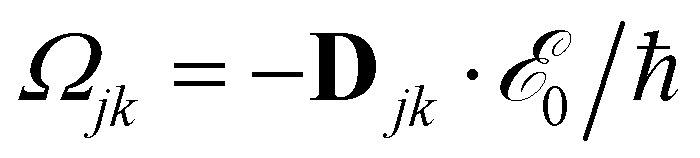
, and *Δ*_*jk*_(*Ω*) = *Ω* − (*ε*_*j*_ − *ε*_*k*_). The population relaxation rate *ℏΓ* and the decoherence rate *ℏγ* are kept fixed to known rates in the target semiconductor (*e.g.*, 27 ps and 27 fs for TiO_2_ surfaces from ref. 95).

The absorbance can be expressed in terms of the stationary populations as^11,92,93,96,97^3
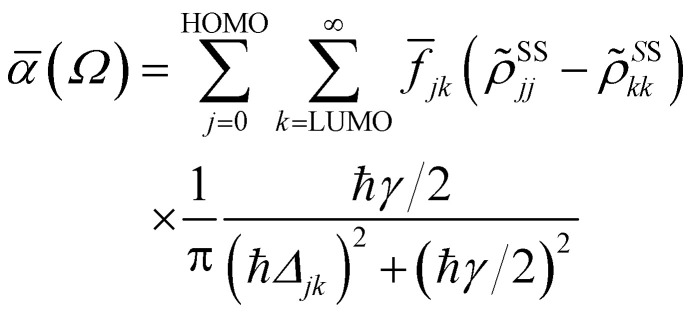
where *f̄*_jk_ is an oscillator strength per active electron.

To investigate the practical applications of materials upon sunlight exposition, the absorbance from eqn (3) is weighted with the absorption of solar energy^88^ (referred to as ‘solar flux’). The solar flux absorption spectrum (*i.e.*, the absorbed flux of light energy) is given as:^92^
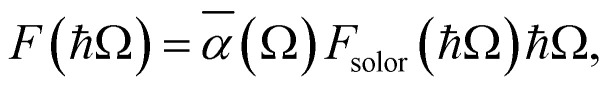
where the solar flux *F*_solar_ is given from the black-body flux distribution of photon energies, normalized to an incident photon flux of 1 kW m^−2^,
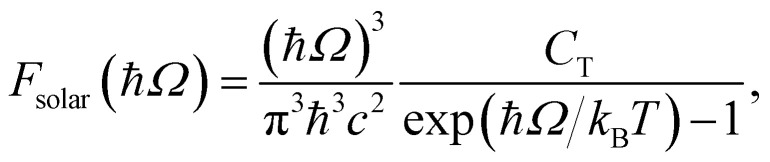
with *C*_T_ as the flux normalization constant. Hence, the incident photon energy flux is simply *F*_solar_(*ℏΩ*)*ℏΩ*.

### 2.4 Computational protocol

The procedure followed in the VASP calculations is briefly outlined as follows: (1) structural optimizations are initially carried out with the DFT-D3 scheme;^98,99^ (2) in the next step, the obtained structures are re-optimized applying the Hubbard DFT + *U* term,^100^ with the spin-polarization accounted for; (3) the optimized structures at DFT + *U*/D3 level are then used in subsequent calculations with the HSE06 hybrid functional of Heyd, Scuseria and Ernzerhof;^101,102^ (4) finally, the orbitals obtained at HSE06 level are utilized for the calculations of the photo-absorption spectra. The HSE06 functional is particularly suitable as it provides values for the band-gap and mid-gap states of semiconductors that agree with experimental observations. For example, it estimates the band-gap of rutile TiO_2_(110) at 3.3 eV, which is consistent with the experimental value of 3.3 ± 0.5 eV (ref. 103).

## 3 Results and discussion

### 3.1 Optical properties of non-encapsulated Cu_5_–TiO_2_ nanoparticles

**Fig. 2:**
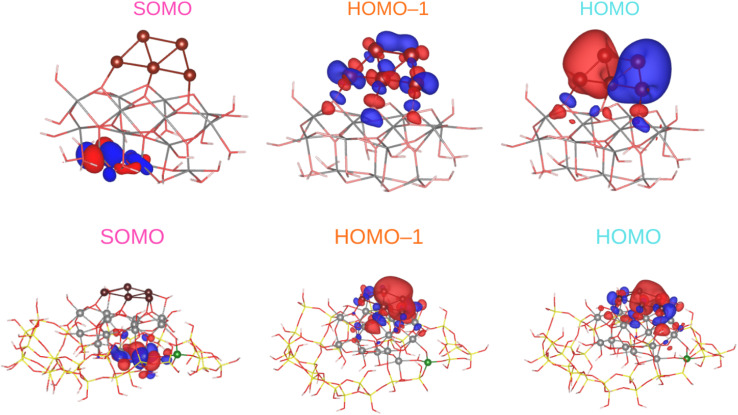
Picture showing isosurfaces of the frontier “single-occupied” (or occupied only for a single spin component) orbital (referred to as SOMO) as well as the highest-energy and second “doubled-occupied” (or occupied for two spin components) molecular orbitals (referred to as HOMO and HOMO-1) of non-encapsulated (upper panel) and zeolite-encapsulated Cu_5_–TiO_2_ nanoparticles (bottom panel).

**Fig. 3:**
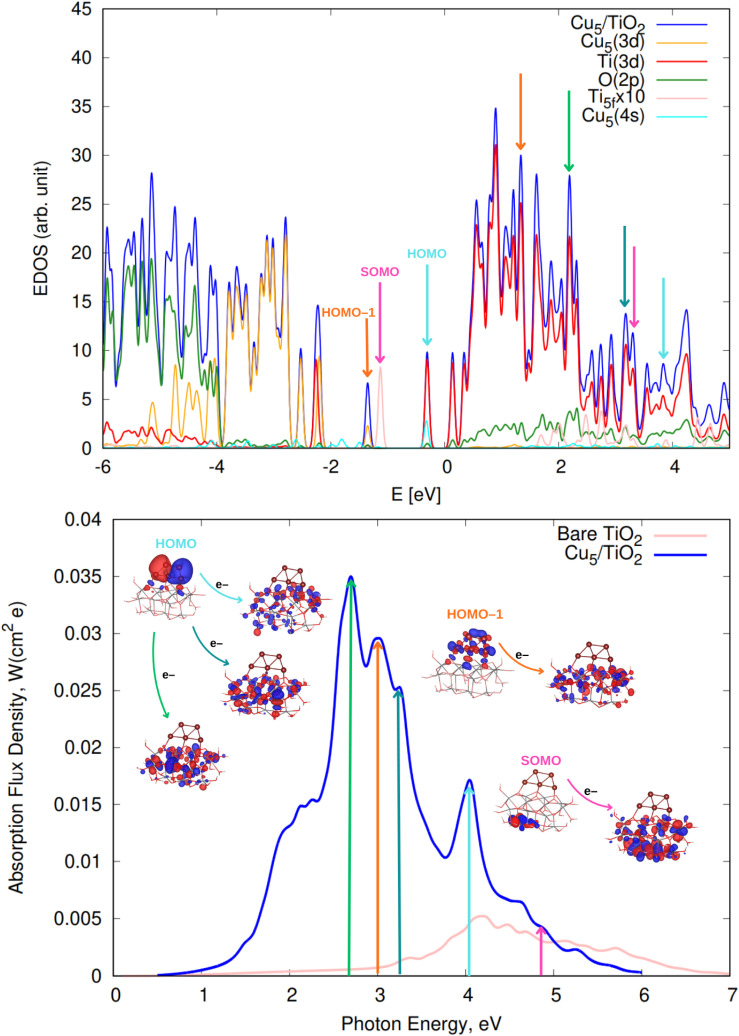
(Upper panel) Electronic density of states (EDOS) of non-encapsulated Cu_5_–TiO_2_ nanoparticles. The projected density of states onto atomic Ti(3d) and O(2p) orbitals as well as the sum of projections of Cu(3d) and Cu(4s) orbitals are also shown along with the projection onto the 3d orbital of the Ti atom hosting the small polaron-like state (*i.e.*, the SOMO state). The arrows indicate the positions of the orbitals responsible for the most intense photo-absorption peaks. (Bottom panel) Photo-absorption spectra of non-encapsulated Cu_5_–TiO_2_ nanoparticles and bare TiO_2_ rutile (110) from ref. 11. The positions of the most intense peaks is also indicated. The insets present the orbitals involved in the photo-excitation processes. The zero of energy is set to the energy of the lowest unoccupied molecular orbital (LUMO).

We begin by discussing the optical properties of Cu_5_-decorated TiO_2_ nanoparticles. To characterize the main features of Cu_5_ adsorption onto the TiO_2_ nanoparticle in the electronic ground state, Fig. 2 presents the frontier orbitals of the structure pictured in Fig. 1 (see also Fig. S1 of the ESI†). The photo-absorption spectra and the projected density of states are displayed in Fig. 3 instead.

#### 3.1.1 Adsorption of Cu_5_ cluster in the TiO_2_ nanoparticle

As a first step, the optimized geometry of the planar trapezoidal Cu_5_-2D structure was taken from ref. 104. This previous study demonstrated that optimized structures of Cu_5_ clusters at DFT-D3 level agree very well with those obtained at higher levels of *ab initio* theory. Thus, the Cu–Cu distances (within 0.05 Å) closely followed those obtained with the coupled cluster method with single, double, and perturbative triple excitations [CCSD(T)]. Keeping this geometry constrained, we inserted the Cu_5_ cluster into the surface of the TiO_2_ nanoparticle, and performed periodic DFT-D3 and DFT + *U*/D3 calculations. Similar to the case of Cu_5_ deposition onto TiO_2_ rutile (110), the Cu_5_ cluster becomes well anchored to the TiO_2_ nanoparticle *via* the basal Cu atoms of the trapezoidal structure, which bind to the bridging oxygen atoms (see Fig. 1). Additionally, the Cu_5_ cluster donates its unpaired electron to the TiO_2_ nanoparticle, leading to the formation of a small polaron-like Ti^3+^ 3d^1^ state (SOMO orbital in Fig. 2) and a concomitant lost of magnetization. However, in contrast to the Cu_5_-induced surface polaron observed in extended TiO_2_ rutile (110) in ref. 11, the unpaired electron becomes localized at a Ti atom within the subsurface region.

The estimate of the interaction energy at the DFT + *U*/D3 level (*ca.* −3.8 eV, see Table 1) indicates a high stability of the Cu_5_ cluster on the small TiO_2_ structure. This value is comparable to the interaction energy reported for the Cu_5_–TiO_2_(110) system (*ca.* −3.5 eV in ref. 11). A recent study involving the open-shell Cu_3_ cluster interacting with a molecular graphene model (coronene) highlighted that the Grimme D3 correction overestimates the dispersion contribution to the cluster–support interaction energy (see ref. 86 for details). However, the DFT-D3 scheme has also been shown to deliver sensible results for van-der-Waals-dominated interactions, such as the interaction of the closed-shell Ag_2_ cluster with graphene^105^ and rutile TiO_2_ (110) surfaces.^96^ The results were consistent with those obtained using, *e.g.*, symmetry adapted perturbation theory (SAPT) with a DFT description of the monomers (*i.e.*, the SAPT(DFT) approach^106,107^) as well as the domain-based local pair natural orbital DLPNO-CCSD(T) method (agreeing to within 10%). Benchmarking the intermolecular Cu_5_–TiO_2_ interaction could benefit from applying the open-shell coupled UMP2 (UMP2C) theory proposed in ref. 86 for transition metal oxides. This approach replaces the uncoupled Hartree–Fock dispersion energy with a coupled dispersion contribution from frequency-integrated linear-response functions in time-dependent unrestricted Hartree–Fock theory. In this work, its application to the interaction potential between a single Cu atom and a TiO_5_H_6_ cluster (see Fig. S2 and Table S4 of the ESI†) shows that the DFT-D3 approach overestimates the interaction energy at the potential minimum by 0.2 eV (*ca.* −0.8 *vs.* −0.6, see Table S4†) and predicts an optimized Cu–TiO_5_H_6_ distance approximately 10% shorter than the UMP2C estimate. Once again, however, as can be observed in Table S2 of the ESI,† the interatomic Cu–Cu distances for the free Cu_5_–TiO_2_ nanoparticle differ minimally from those reported using high level *ab initio* theory in ref. 104. In contrast, the range of inter-atomic Cu–O distances aligns well with those reported in DFT studies on the structural and magnetic properties of low-index Cu_2_O surfaces.^108^

**Table 1:** Cu_5_–TiO_2_ nanoparticle and Cu_5_–TiO_2_@zeolite interaction energies calculated with DFT-D3 approach using the all-electron approach with the ORCA code (referred to as DFT-D3, left-hand column) and the DFT + *U*/D3 approach (right-hand column), including the Hubbard DFT + *U* correction, in 3d4s + PP calculations with the VASP code (see Methods section). Values are presented in eV

	DFT-D3//all-electron	DFT + *U*/D3//3d4s + PP
Cu_5_–TiO_2_	−3.26	−3.79
Cu_5_–TiO_2_@zeolite	−3.51	−3.93

As shown in the middle panel of Fig. 2, the HOMO-1 features a clear hybridization between Cu(3d) orbitals and O(2p) orbitals of the nearest bridging oxygen atoms (see also Fig. S1 of the ESI†), indicating a covalent nature of the Cu_5_–TiO_2_ bonding. The energy of the HOMO-1 in the free Cu_5_ cluster is very close to the HOMO energy of the free TiO_2_ nanoparticle, facilitating the mixing of Cu(3d) and O(2p) contributions. In contrast, the HOMO, which is single occupied and dominated by 4s contributions from all Cu atoms in the free cluster, becomes double occupied and dominated by 4s contributions localized on the two terminal apical Cu atoms of the trapezoidal structure. This outcome suggests an internal charge transfer from the basal Cu atoms to the apical ones, leading to a polarization of the electronic charge within the trapezoidal structure. This polarization tilts the structure toward an uncoordinated five-fold (5f) Ti atom on the surface of the TiO_2_ nanoparticle. As shown in the projected density of states (Fig. 3), this orbital also presents Ti(3d) contributions.

Interestingly, in all-electron (non-periodic) calculations excluding the DFT + *U* Hubbard term (see Fig. S1 of the ESI†), the frontier orbitals are very similar to those obtained in periodic calculations. In both cases, the Cu_5_ cluster loses its magnetic moment in favor of the SOMO which characterizes the localization of its unpaired electron in the 3d orbital of a Ti atom. A comparison of periodic calculations using large (4-valence electrons 3d4s) and small (12-valence electrons 3s3p3d4s) pseudopotentials for Ti atoms also indicates a modest impact on the frontier orbitals. Both total and average Bader charges on Cu atoms (see Table S1 of the ESI†) differ only slightly (to within 0.1 and 0.02|*e*|). Larger variations are observed in the Bader charges of the Ti atoms and O–H species of the TiO_2_ nanoparticle (*e.g.*, up to 24% for Ti atoms). Appealing, Mulliken analysis^109^ indicates that the Cu_5_ cluster becomes slightly negatively charged (by −0.2|*e*| in contrast to the Bader analysis, which assigns a total charge of 0.8|*e*|). Such discrepancies between Mulliken and Bader charges for transition metal atoms have been previously observed.^110^ This outcome indicates the high degree of covalent character in the Cu_5_–TiO_2_ chemical bonding and suggests a charge-transfer mechanism where the TiO_2_ nanoparticle supplies charge to Cu_5_ through the Cu–O bonds. Additionally, there is also back transfer of charge from the TiO_2_ nanoparticle to the d-type orbitals centered on the Cu atoms, which in turn, become mixed with (primarily) O(2p) orbitals. This mechanism also explains why the total Bader charge of Cu_5_ (−0.8|*e*|) is less than unity. Similarly, when charges are estimated based on projections of one-electron-wavefunctions from VASP calculations onto spherical harmonics centered on Cu atoms, the Cu atoms are predicted to be slightly, yet negatively, charged. However, unlike the Bader charges, these projected charges show a significant dependence on the pseudopotential used for the Ti atoms.

#### 3.1.2 Photo-absorption spectrum of the Cu_5_–TiO_2_ composite nanoparticle

Having analyzed the Cu_5_-decorated TiO_2_ nanoparticle in the ground electronic state, we now focus on its optical excitation. Fig. 3 (bottom panel) illustrates how the photo-absorption spectra exhibit the higher intensity peaks in the short-wave visible region (between 2.7 and 3.2 eV). The projected electronic density of states (EDOS) indicates that the main peaks primarily originate from direct photoinduced transfer of electrons from the frontier orbitals HOMO-1 and HOMO. These orbitals are mainly composed of Cu(3d) and Cu(4s) atomic contributions, and bearing chemical mixing with O(2p) and Ti(3d) orbitals (see also the pictures of the orbital densities in the insets of Fig. 3). A single electron ‘jumping’ from the HOMO to Ti(3d) states also gives rise to a high intensity peak at the medium-wave UV region (indicated with a cyan arrow in Fig. 3). This peak is very close to the corresponding peak in the spectrum of bare TiO_2_ (shown in pink in the bottom panel of Fig. 3). However, as discussed in ref. 11, for bare TiO_2_, the peak arises from electron transfer originating in O(2p) orbitals and transitioning to 3d orbitals of surface and subsurface atoms.

There is also photo-induced electron transfer from the (SOMO) polaron-like state (indicated with a pink arrow in Fig. 3) but the associated absorption peak is much less intense and located in the short-wave UV region (*ca.* 4.9 eV). As shown in Fig. 3, the photo-excitation leads to the delocalization of the polaronic charge across all Ti atoms of the nanoparticle. In contrast, for Cu_5_-modified TiO_2_(110) surfaces,^11^ surface polarons are formed. Upon photo-excitation with visible light, these polarons become delocalized over all surface Ti atoms (*i.e.*, indicating the photo-induced conversion of a small localized polaron into a large delocalized polaron^2,111,112^). Moreover, for Cu_5_-decorated TiO_2_ nanoparticles, the spectra clearly peak at the visible region whereas for the extended Cu_5_-modified TiO_2_(110) system,^11^ the peak at the UV region is still the most intense. As discussed in ref. 11, Cu_5_ clusters enhance the absorption in the UV region through a mechanism involving electron transfer from surface and subsurface O anions to surface and subsurface Ti atoms. The number of Ti atoms and O anions in the unit cell for the TiO_2_ nanoparticle is much lower than for the extended TiO_2_(110) surface, explaining why the UV peak is not the more intense for the nanoparticle.

#### 3.1.3 CO_2_ photo-reduction on Cu_5_–TiO_2_ nanoparticles

**Fig. 4:**
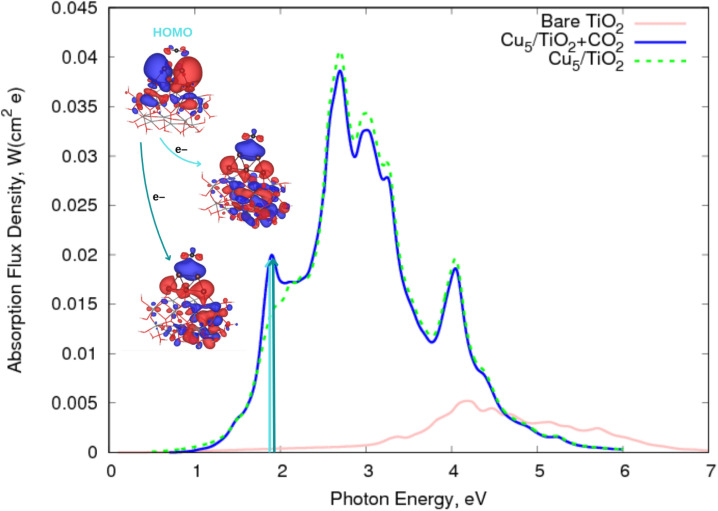
Photo-absorption spectra of non-encapsulated Cu_5_–TiO_2_ nanoparticles (with and without CO_2_ adsorbed) and bare TiO_2_ rutile (110) from ref. 11.

As highlighted in ref. 113 and 20, Cu_5_-decorated TiO_2_ nanoparticles are capable of adsorbing CO_2_ molecules in a physisorption state. Fig. 4 shows that the p-type orbitals of the oxygen atoms in the CO_2_ molecule contribute to the highest doubly occupied orbital of the system (HOMO). Upon visible light irradiation (about 1.9 eV), an electron is transferred from the HOMO to two acceptor orbitals whose energies differ by less than 0.05 eV due to the degeneracy of the p-type orbitals centered on the O atoms of the CO_2_ molecule. However, because of a symmetry breaking of the electronic structure caused by formation of a polaron-like state at one side of the TiO_2_ nanoparticle, these orbitals are no longer completely degenerate. The photo-induced electron transfers generate a CO_2_˙^−^ radical bearing weakened C=O bonds compared to those featured by the neutral CO_2_ counterpart, facilitating its cleavage and dissociation. Otherwise, the physisorption of the CO_2_ molecule on top of the Cu_5_ cluster modifies the spectrum profile of the bare Cu_5_–TiO_2_ nanoparticle only slightly. Once again, the major modification is observed at about 1.9 eV (blue and green arrows in Fig. 4), with the transition responsible for the two additional peaks involving the electron “jump” from the HOMO to final states characterized as the CO_2_˙^−^ attached to the Cu_5_–TiO_2_ nanoparticle.

### 3.2 Optical properties of zeolite-encapsulated Cu_5_–TiO_2_ nanoparticles

In the first part of this section, the geometries and the electronic structure of free and zeolite-encapsulated Cu_5_–TiO_2_ nanoparticles are compared, as obtained using the DFT + *U*/D3 approach. In the second section, the frontier orbitals and the photo-absorption spectrum of the zeolite-encapsulated Cu_5_–TiO_2_ nanoparticle are analyzed based on final HSE06 calculations.

#### 3.2.1 Encapsulation effects in the structure and Bader charges of the Cu_5_–TiO_2_ composite nanoparticle

Let us now focus on the effects of the encapsulation of the Cu_5_–TiO_2_ nanoparticle within the zeolite micropores, as shown in Fig. 1. Inter-atomic Cu–Cu, shortest Cu–O distances and Bader charges are displayed in Tables S1 and S2 of the ESI.† As shown in Table 1, the interaction energy between Cu_5_ and TiO_2_ increases slightly upon encapsulation of the latter (−3.79 *vs.* −3.93 eV). Notably, the initial ‘stay-up’ planar trapezoidal Cu_5_ structure transforms into a ‘flat-lying’ isomer upon encapsulation. This behaviour is partially driven by electrostatic interactions. As discussed in ref. 64, the substitution of Si by Al in the zeolite framework introduces an additional negative electronic charge, which is compensated by an extra donation from the Cu_5_ cluster. Consequently, the total Bader charge increases from 0.8 to 1.4|*e*| (see Table S1 of the ESI†). To enable this charge compensation, the two apical Cu atoms of the trapezoidal Cu_5_ cluster bond with the terminal in-plane oxygen anions of TiO_2_ nanoparticle (see Fig. 1). This interaction significantly elongates the Ti–O bonds toward the two Cu atoms, causing Cu_5_ to lie flat on the TiO_2_ nanoparticle's surface. This is clearly reflected in the decrease of the shortest Cu–O distances from 4.3–4.5 to *ca.* 2 Å. Additionally, the asymmetric position of the Al atom within the zeolite cage distorts the planar trapezoidal Cu_5_-2D structure, resulting in significantly elongated Cu–Cu bonds compared to those in the non-encapsulated Cu_5_–TiO_2_ composite nanoparticle (see Table 1). In contrast, the non-encapsulated system lacks this asymmetry, preserving the symmetry of the 2D-Cu_5_ structure upon deposition on the free TiO_2_ nanoparticle. It is also remarkable that two different pseudopotentials yield very similar Bader charges (differing by 7% at most). Moreover, upon encapsulation, the Bader charges of species other than Cu atoms differ by less than 6%. Furthermore, the total dipole moment vector from Table S5 of the ESI† (relative to the center-of-mass and the geometrical center), shows that the distribution of charges on the Cu_5_–TiO_2_*@*zeolite structure using the HSE06 functional is rather asymmetric, with the vector pointing away from the center at: (−7.74, −32.01, 62.26)|*e*| Å. This uneven electron density distribution is further illustrated in Fig. S3 of the ESI.† The large total molecular dipole moment (70.43) indicates a strong potential influence on the structure's interactions with external electric fields and nearby molecules.^114^

As illustrated in Fig. 2 (bottom panel), the electronic structure of the composite Cu_5_–TiO_2_ nanoparticle undergoes modification upon encapsulation, accompanied by aforementioned structural changes. The polaron-like electron relocates to a Ti atom covalently bonded to an Al atom within the zeolite framework. The SOMO, which exhibits polaron-like characteristics, becomes the highest occupied molecular orbital, in contrast to its position as the second-highest orbital in the non-encapsulated system. The doubly-occupied HOMO involves s-type orbitals from the Cu cluster and p-type orbitals from the oxygen atoms, including those from the additional OH groups of TiO_2_. The next frontier orbital, HOMO-1, remains localized at the nanoparticle, specifically at the bonding site with the Al atom. HOMO-1 features Cu d-type orbitals and O p-type orbitals but also includes Cu s-type orbitals.

Our conclusions align closely with those reported in a DFT-based study investigating the influence of zeolite supports on the catalytic properties of Cu_5_ clusters.^64^ In that study, it was observed that substituting a single Si atom with Al in the CHA zeolite generates a net negative charge, which is compensated by the appearance of a positive Bader charge (*ca.* 0.8|*e*|) on the Cu_5_ clusters. This charge redistribution leads to a reduction in the shortest Cu–O distances to 1.974 Å. This value is comparable to those obtained for Cu_5_–TiO_2_ within the zeolite cavity (see Table S2 of the ESI†). Similarly, the planar Cu_5_ cluster undergoes structural distortion within the micropores of the CHA zeolite,^64^ with the shortest Cu–O distance being comparable to that observed in the zeolite@TiO_2_ system (1.9 *vs.* 2.0 Å, see Table S1 of the ESI† and ref. 64).

#### 3.2.2 Photo-absorption spectrum of the zeolite-encapsulated Cu_5_–TiO_2_ composite nanoparticle

**Fig. 5:**
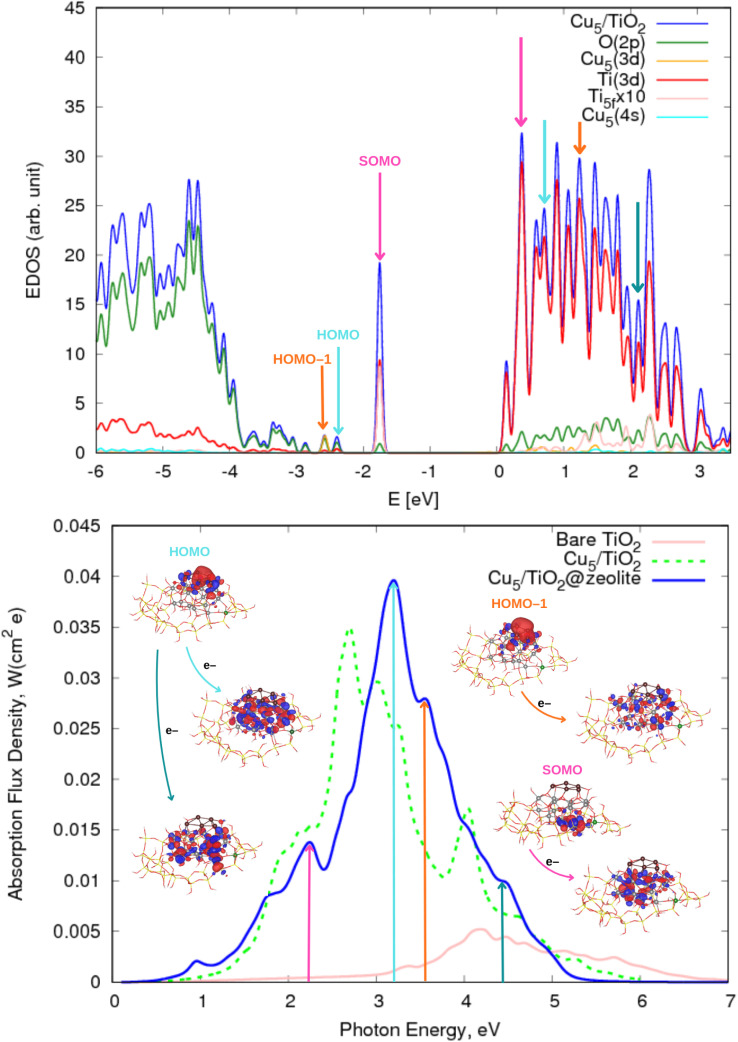
(Upper panel) Electronic density of states (EDOS) of the zeolite-encapsulated Cu_5_–TiO_2_ nanoparticle shown in Fig. 1. The projected density of states onto atomic Ti(3d) and O(2p) orbitals as well as the sum of projections of Cu(3d) and Cu(4s) orbitals are also shown along with the projection onto the 3d orbital of the Ti atom hosting the small polaron-like state (*i.e.*, the SOMO state). The arrows indicate the positions of the orbitals responsible of the most intense photo-absorption peaks. (Bottom panel) Photo-absorption spectra of zeolite-encapsulated and non-encapsulated Cu_5_–TiO_2_ nanoparticles and bare TiO_2_ rutile (110) from ref. 11. The positions of the most intense peaks is also indicated. The insets present the orbitals involved in the photo-excitation processes. The zero of energy is set to the energy of the lowest unoccupied molecular orbital (LUMO).

The analysis of the photo-absorption spectrum and the electronic density of states (EDOS) of the zeolite-encapsulated Cu_5_-decorated TiO_2_ nanoparticle and its comparison with the non-encapsulated counterpart reveal several important similarities and differences. As shown in Fig. 5, both systems exhibit their most intense peak in the visible light region. However, for the encapsulated nanoparticle, this peak is shifted towards the short-wave visible range (around 2.9–3.2 eV). This peak originates from electron transitions from the HOMO, which involves both Cu s- and d-type orbitals, to bands comprising Ti 3d-orbitals and O 2p-orbitals of atoms located in the upper and lower layers of the TiO_2_ structure. Additionally, a significant peak appears in the UV region at approximately 3.6 eV, corresponding to transitions from the HOMO-1. Similarly to the HOMO, the HOMO-1 involves Cu s- and d-type orbitals but also interacts with Ti 3d- and O 2p-orbitals. Interestingly, this transition also engages Cu d- and s-type orbitals that bond with additional oxygen atoms, due to the ‘flat-lying’ arrangement of the copper cluster in the encapsulated system. In the UV region, there is also a transition from the HOMO at *ca.* 4.5 eV. This transition leads to the filling of bands involving Ti d- and O p-orbitals, but interestingly, it also involves the orbitals of oxygen atoms from the zeolite framework. Specifically, these oxygen atoms are in close proximity to the part of the nanoparticle that is covalently bonded to an aluminum atom in the zeolite structure.

The most pronounced difference between the spectra of non-encapsulated and encapsulated nanoparticles, however, lies in the photo-excitation of the SOMO, *i.e.*, the polaron-like state with an electron localized in a Ti(3d) orbital. In the encapsulated nanoparticle, the SOMO is the highest-energy occupied orbital, and the corresponding transition peak is significantly more intense and shifted from the UV region to the long-wave visible region (*ca.* 2.3 eV). The acceptor state is a delocalized polaron-like state involving primarily Ti d- and O p-orbitals from the upper layer of the encapsulated TiO_2_ nanoparticle at the bottom of the conduction band while, for the non-encapsulated system, the accepting state involves Ti(3d) orbitals from the inner layer. On the one hand, as shown in previous works on Ag_5_-decorated TiO_2_ surfaces,^2,111,112^ the photo-excitation of a small electron polaron leads to a large delocalized electron polaron, highlighting its dual nature. On the other hand, the shifting of the delocalized polaron-like state from bottom to top layers upon encapsulation can be explained by considering the net generated charge caused by the substitution of a Si atom by Al in the zeolite structure.

The main findings regarding the photo-absorption spectra can likely be generalized to other zeolite structures with cavities hosting TiO_2_ nanoparticles of appropriate size. As previously discussed for Cu_5_@zeolite composites,^64^ the structure adopted by Cu_5_ clusters may depend on the number of Si atoms replaced by Al ones. However, the photo-absorption spectra are still expected to exhibit the high absorption peaks in the visible region. Notably, both zeolite-encapsulated and non-encapsulated systems demonstrate absorption peaks in the visible range regardless of the specific structure adopted by the Cu_5_ cluster. Further investigation is required to explore the interaction of smaller nanoparticles that can fit into different pore sizes and to examine the influence of multiple aluminum atoms on the physicochemical properties of the cluster-decorated TiO_2_ nanoparticles. The effects of multiple aluminum atoms in zeolite frameworks on bare subnanometric metal clusters have been previously analyzed (see, *e.g.*, ref. 64). Extending these studies to cluster-decorated TiO_2_ nanoparticles could offer new perspectives on their photoactivity and functional properties.

## 4 Conclusions

The transition to a green economy necessitates advancement of industrial chemistry, the reduction of greenhouse gases in the atmosphere and the generation and storage of environmentally clean energy. Theoretical and experimental results have demonstrated how Cu_5_ clusters significantly improve the catalytic, photocatalytic and solar energy conversion properties of titanium dioxide, one of the most widely used materials for this type of process (see, *e.g.*, ref. 20 as a recent perspective). These findings are not only relevant to industrial chemistry or clean energy generation, but also to reducing atmospheric carbon dioxide. In this work, we take a step forward by exploring the possibility of using small TiO_2_ nanoparticles embedded within the micropores of a zeolite structure as a cost-effective alternative to TiO_2_ surfaces or large TiO_2_ nanoparticles for these applications. Moreover, spatial confinement within the zeolites microporous materials allows for the stabilization of Cu_5_ clusters, suppressing their aggregation. Remarkably, we point out three important findings: (1) the high stability of the Cu_5_ clusters on TiO_2_@zeolite structures; (2) the activation of the small TiO_2_ nanoparticles as visible-light photoactive through the adsorption of the Cu_5_ clusters; (3) their applicability in trapping CO_2_ molecules from the atmosphere which, when irradiated with visible light, are transformed into CO_2_˙^−^ radicals. These radicals represent a precursor state for dissociation due to their weakened C–O bonds.

Interestingly, while the photo-absorption spectrum of the Cu_5_–TiO_2_(110) interface^11^ exhibited peaks in both the visible and the UV regions, the zeolite-encapsulated Cu_5_–TiO_2_ nanoparticle shows a pronounced peak in the visible region. This outcome suggests that the zeolite structure not only serves to stabilize the Cu_5_ clusters but also to improve the optical response of TiO_2_ in the visible region as compared to Cu_5_-decorated rutile TiO_2_(110).^11^ Moreover, natural zeolite has been proven to be a good supporting material for TiO_2_ nanoparticles in photo-catalytic reactions due to its high specific surface area, adsorption capacity, stability, hydrophobicity, crystal size, optical absorption properties, lifetime of charge carriers, unique uniform pores and channel sizes, *etc.* (see, *e.g.*, ref. 115–120), with the applications including water waste treatment.^121,122^ Our findings also suggest the possibility of shifting experimentally demonstrated photocatalytic reactions such as oxidation of propene and CO_2_ photo-reduction with TiO_2_ nanoparticles filling the zeolite cavities^117^ and metal–organic framework mesospores^123^ with UV light towards the visible *via* their decoration with Cu_5_ clusters.

## Data availability

The data supporting the findings of this study are available in the ESI of this article.†

## Author contributions

Conceptualization: M. P. d. L.-C.; investigation: K. M. K, L. L. C., and M. P. d. L.-C.; methodology: K. M. K. and M. P. d. L.-C.; data analysis: K. M. K. and M. P. d. L.-C.; data curation: K. M. K. and M. P. d. L.-C.; visualization: K. M. K. and M. P. d. L.-C.; supervision: M. P. d. L.-C.; project administration: M. P. d. L.-C.; funding acquisition: M. P. d. L.-C.; software: K. M. K., L. L. C., and M. P. d. L.-C.; writing: K. M. K and M. P. d. L.-C.; revision and editing: K. M. K., L. L. C., and M. P. d. L.-C.

## Conflicts of interest

There are no conflicts to declare.

## Supplementary Material

RA-015-D4RA08633C-s001
