# Supplementary material for: Functionalization of zeolite-encapsulated Cu5 clusters as visible-light photoactive sub-nanomaterials
Source: RSC Adv. 2025 Jan 22;15(3):2086–98. doi: 10.1039/d4ra08633c (PMC11753201; doi:10.1039/d4ra08633c)
Supplement: RA-015-D4RA08633C-s001 [file RA-015-D4RA08633C-s001.pdf]

Supplementary Information (SI) for RSC Advances.
This journal is © The Royal Society of Chemistry 2025

RSC Advances

PAPER

Cite this: DOI: 00.0000/xxxxxxxxxx

Electronic Supplementary Information: Functionalization of zeolite–encapsulated Cu_5 clusters as visible–light photoactive sub–nanomaterials

Katarzyna M. Krupka,[a] Lenard. L. Carroll,[a] and María Pilar de Lara-Castells*[a]

Notes and references

1 T. Lu, *J. Chem. Phys,*, 2024, **161**, 082503.
2 T. Lu and F. Chen, *J. Comp. Chem.*, 2011, **33**, 580–592.
3 K. M. Krupka, A. Krzemińska and M. P. de Lara-Castells, *RSC Advances*, 2024, **14**, 31348–31359.

[a] *Institute of Fundamental Physics (AbinitSim Unit, ABINITFOT Group), Madrid, Spain. E-mail: Pilar.deLara.Castells@csic.es*

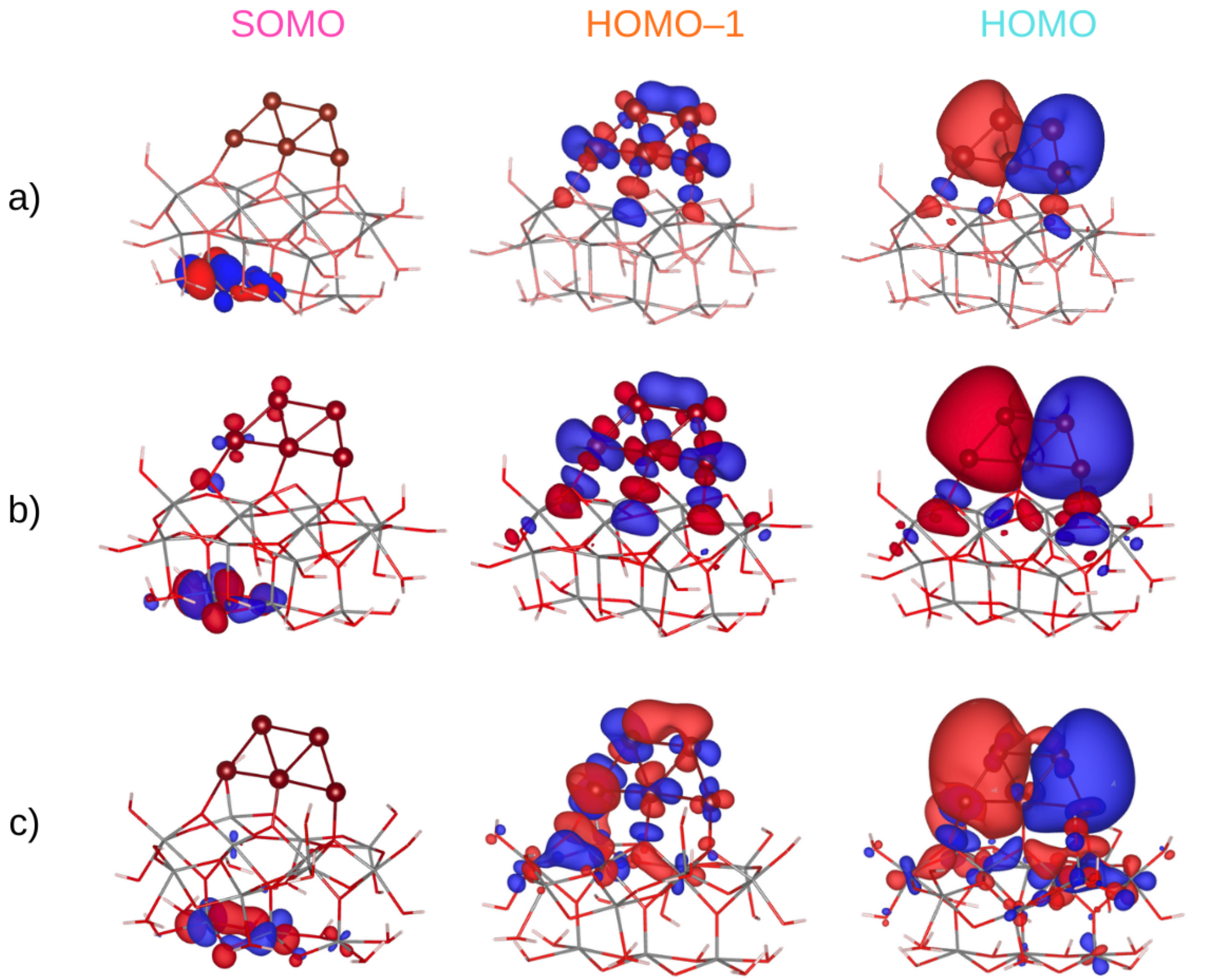

Fig. S1 Picture showing isosurfaces of the frontier "single-occupied" (or occupied only for a single spin component) orbital (referred to as SOMO) as well as the highest-energy and second "doubly-occupied" (or occupied for two spin components) molecular orbitals (referred to as HOMO and HOMO–1) of non-encapsulated Cu_5–TiO_2 nanoparticles. Calculations: a) DFT+U/D3 with small (12-valence electrons $3s3p3d4s$) pseudopotential for Ti atoms, b) DFT+U/D3 with large (4-valence electrons $3d4s$) pseudopotential for Ti atoms, c) all-electron DFT-D3/aug-cc-pVTZ

Type of atom	Sum of Bader charges			Average Bader charge		
	Zeolite–encapsulated					
	$3s3p3d4s$+PP for Ti	$3d4s$+PP for Ti	all–electron	$3s3p3d4s$+PP for Ti	$3d4s$+PP for Ti	all–electron
Cu	1.41	1.31	1.43	0.28	0.26	0.29
Ti	30.15	30.22	27.68	2.32	2.32	2.13
O	–16.33	–16.46	–14.14	–1.36	–1.37	–1.18
OH	–15.28	–15.61	–14.25	–0.54	–0.56	–0.51
	Non–encapsulated					
Type of atom	$3s3p3d4s$+PP for Ti	$3d4s$+PP for Ti	all–electron	$3s3p3d4s$+PP for Ti	$3d4s$+PP for Ti	all–electron
Cu	0.82	0.72	0.82	0.16	0.14	0.16
Ti	30.27	30.85	27.80	2.33	2.37	2.14
O	–16.83	–17.02	–14.52	–1.40	–1.42	–1.21
OH	–14.25	–14.85	–14.11	–0.51	–0.53	–0.50

Table S1 Sums of Bader charges and average Bader charge of each type of atom. O refers to the oxygen atoms not involved in O–H covalent bonds, O(H) refers to the oxygen atoms involved in O–H covalent bonds, OH indicates Bader charges of the –OH groups.[1,2]

Distance Label	Distance encapsulated (Å)	Distance non–encapsulated (Å)	Distance non–encapsulated (Å) (alt.)
Cu_1-Cu_2	2.377	2.440	3.068
Cu_1-Cu_4	2.416	2.291	2.427
Cu_2-Cu_3	2.668	2.436	2.349
Cu_2-Cu_4	2.451	2.338	2.484
Cu_2-Cu_5	2.313	2.335	2.382
Cu_3-Cu_5	2.652	2.309	2.416
Cu_4-Cu_5	2.335	2.560	2.604
Cu_1-O_N	1.898	1.927	2.061
Cu_2-O_N	1.933	1.946	1.969
Cu_3-O_N	1.859	1.916	2.001
Cu_4-O_N	1.972	4.362	1.975
Cu_5-O_N	1.986	4.524	2.087

Table S2 Cu–Cu and Cu–O_N distances from the zeolite-encapsulated and non-encapsulated Cu_5/TiO_2 structures (including an alternative structure based on the optimized structure of Cu_5/TiO_2@Zeolite). O_N represents the nearest oxygen atom to the Cu_5 cluster. Atomic labels are from Figure 1 in the main manuscript.

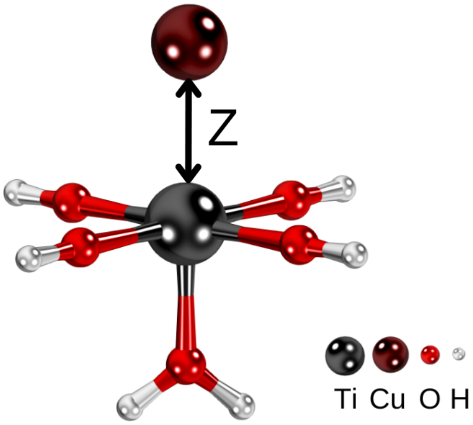

Fig. S2 Cu–TiO_5H_6 complex used for benchmark study. Z is defined as the distance between the Cu and Ti atoms.

Distance (Z) [Å]	DFT–D3 [eV]	UMP2C [eV]
2.00	–0.20	1.83
2.25	–0.50	0.92
2.50	–0.8	0.00
2.70	–0.78	–0.44
3.00	–0.64	–0.57
3.50	–0.42	–0.45
4.00	–0.28	–0.34
4.50	–0.19	–0.24
5.00	–0.13	–0.18
5.50	–0.07	–0.12
6.00	–0.05	–0.07

Table S3 Numerical values of Cu–TiO_5H_6 interaction energies (in eV) at DFT-D3 and UMP2C[3] levels of theory with the aug-cc-pVTZ basis set.

System	Dipole Moment Vector
HSE06 ($3d4s$+PP for Ti) (Total)	(–7.74, –32.01, 62.26)
HSE06 ($3d4s$+PP for Ti) (Origin) (Cu_5)	(–69.39, –61.51, –119.19)
HSE06 ($3d4s$+PP for Ti) (Center of Mass) (Cu_5)	(–8.14, –4.02, 15.30)
HSE06 ($3d4s$+PP for Ti) (Center of Geometry) (Cu_5)	(–8.61, –4.72, 16.87)

Table S4 Dipole moment vectors for Cu_5/TiO_2@zeolite calculated with the HSE06 functional large (4-valence electrons $3d4s$) pseudopotential for Ti atoms. Calculations based on the orbital projection charges, with the dipole moment relative to the center of mass, origin and center of geometry for Cu_5.

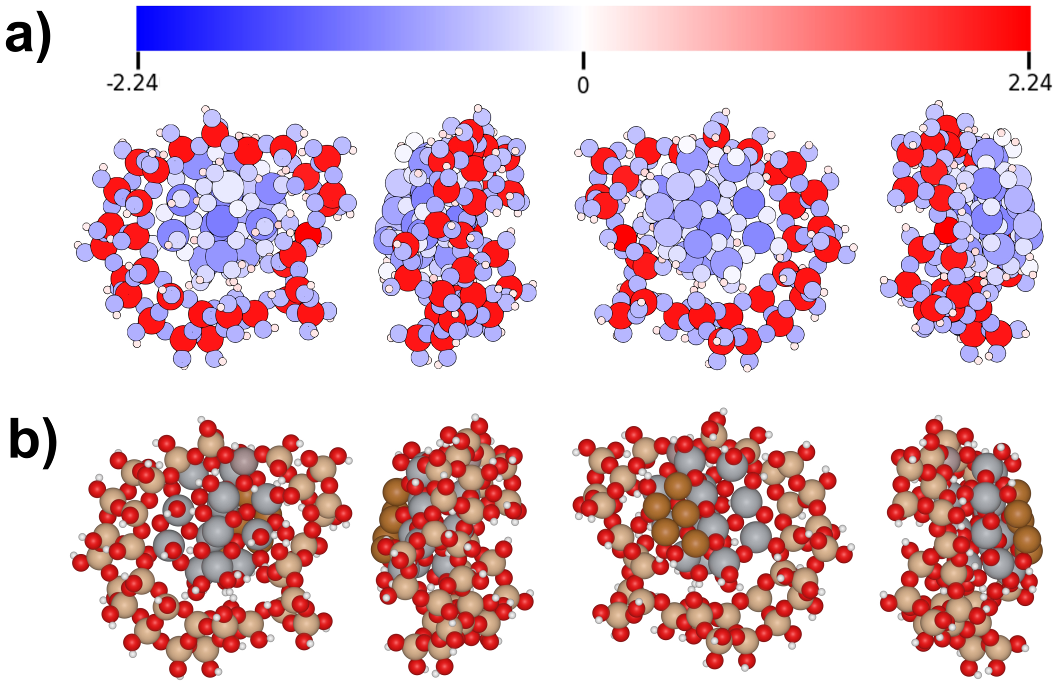

Fig. S3 a) Orbital projection charge color plot of Cu_5/TiO_2@zeolite calculated with the HSE06 functional with large (4-valence electrons $3d4s$) pseudopotential for Ti atoms. b) From the second row of structures, Cu = Bronze, O = Red, H = White, Ti = Silver Sand, Si = Desert Sand, Al = Silver Pink.
